# RETRACTED: Luo et al. Polyphyllin I Promotes Autophagic Cell Death and Apoptosis of Colon Cancer Cells via the ROS-Inhibited AKT/mTOR Pathway. *Int. J. Mol. Sci.* 2022, *23*, 9368

**DOI:** 10.3390/ijms262311528

**Published:** 2025-11-28

**Authors:** Qihui Luo, Lanlan Jia, Chao Huang, Qi Qi, Asad Jahangir, Yu Xia, Wentao Liu, Riyi Shi, Li Tang, Zhengli Chen

**Affiliations:** 1 Laboratory of Experimental Animal Disease Model, College of Veterinary Medicine, Sichuan Agricultural University, Chengdu 611130, China; lqhbiology@163.com (Q.L.);; 2 Key Laboratory of Animal Disease and Human Health of Sichuan Province, College of Veterinary Medicine, Sichuan Agricultural University, Chengdu 611130, China; 3 Center for Paralysis Research & Department of Basic Medical Sciences, College of Veterinary Medicine, Purdue University, West Lafayette, IN 47907, USA

The journal retracts the article titled “Polyphyllin I Promotes Autophagic Cell Death and Apoptosis of Colon Cancer Cells via the ROS-Inhibited AKT/mTOR Pathway” [1], cited above.

Following publication, the authors contacted the Editorial Office to raise concerns relating to Figure 2A(a) and Figure 2B(a), which appears to be the same image. As Figure 2A(a) and Figure 2B(a) were both without PPI treatment, we misused the images when we disposed of the data of this manuscript.

Adhering to our standard procedure, the Editorial Office and Editorial Board conducted an investigation that confirmed Figure 2A(a) and Figure 2B(a) are the same image. As a result, the Editorial Office, Editorial Board, and authors have decided to retract this article as per MDPI’s retraction policy (https://www.mdpi.com/ethics#_bookmark30, accessed on 1 November 2025).

This retraction was approved by the Editor-in-Chief of the *IJMS* journal.

The authors agreed to this retraction.
